# Boosting Thermoelectric Performance of Bi_2_Te_3_ Material by Microstructure Engineering

**DOI:** 10.1002/advs.202308056

**Published:** 2023-12-07

**Authors:** Guoxiang Wang, Fanzheng Meng, Yingqi Chen, Andriy Lotnyk, Xiang Shen

**Affiliations:** ^1^ Laboratory of Infrared Materials and Devices The Research Institute of Advanced Technologies Ningbo University Ningbo Zhejiang 315211 China; ^2^ Leibniz Institute of Surface Engineering (IOM) Permoserstr. 15 D‐04318 Leipzig Germany; ^3^ Institute of Ocean Engineering Ningbo University Ningbo Zhejiang 315211 China

**Keywords:** microstructure, multilayer, power factor, thermoelectric film

## Abstract

Due to the intrinsic contradiction of electrical conductivity and Seebeck coefficient in thermoelectric materials, the enhancement for the power factor (*PF*) is limited. Since the *PF* decides the output power, strategies to the enhancement of *PF* are of paramount importance. In this work, Bi_2_Te_3_/Sb and Bi_2_Te_3_/W multilayer films are proposed to enhance the thermoelectric properties. Both systems possess extremely high conductivity of ≈5.6 × 10^5^ S m^−1^. Moreover, the electrical conductivity and Seebeck coefficient simultaneously increase as temperature rising, showing the overcome of the intrinsic contradiction. This results in ultrahigh *PF*s of 1785 µWm^−1^ K^−2^ for Bi_2_Te_3_/W and of 1566 µWm^−1^ K^−2^ for Bi_2_Te_3_/Sb at 600 K. Thermal heating of the Bi_2_Te_3_/Sb multilayer system shows compositional changes with subsequent formation of Bi‐Te‐Sb phases, Sb‐rich Bi‐Te precipitates, and cavities. Contrary, the multilayer structure of the Bi_2_Te_3_/W films is maintained, while Bi_2_Te_3_ grains of high‐crystalline quality are confined between the W layers. In addition, bilayer defects in Bi_2_Te_3_ and smaller cavities at the interface to W layers are also observed. Thus, compositional and confinement effects as well as structural defects result in the ultrahigh *PF*. Overall, this work demonstrates the strategies on how to obtain ultrahigh *PF*s of commercial Bi_2_Te_3_ material by microstructure engineering using multilayer structures.

## Graphical abstract

The interfacial effect of Bi_2_Te_3_/Sb and Bi_2_Te_3_/W multilayer thin films disrupts the traditional trade‐off between Seebeck coefficient and conductivity, resulting in exceptionally high power factor (PF) values of 1785 μW mK^−2^ for Bi_2_Te_3_/W and 1566 μW mK^−2^ for Bi_2_Te_3_/Sb at 600 K. The compositional and confinement effects as well as structural defects contribute to the remarkable increase in PF.

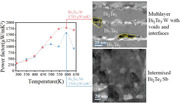

## 1 Introduction

The invention of wearable devices and micro‐electronics has raised the demand for sustainable power supply that can be integrated within the electronic systems to generate power.^[^ ^1^ ^]^ Self‐powered energy supplies can largely satisfy the requirement.^[^ ^2^ ^]^ Conventional methods for power manufacturing have difficulties in their miniaturization.^[^ ^3^ ^]^ In the last decades, thermoelectric (*TE*) generators have attracted considerable attention due to their unique capability of converting heat into electrical power directly.^[^ ^4^ ^]^ Moreover, *TE* thin films can overcome current issues of bulk *TE* powders as they are easy to be integrated into micro‐scale systems.^[^ ^5^ ^]^ The energy conversion property of *TE* devices is valued by the dimensionless *TE* figure of merit *ZT* = *S* ^2^ *σTκ* ^−1^, where *S*, *σ*, *κ* and *T* are the Seebeck coefficient, electrical conductivity, thermal conductivity and absolute temperature, respectively. The properties of *TE* devices greatly rely on the inherent properties of *TE* materials.^[^ ^6^ ^]^ Particularly, power factor (*PF*) (*S* ^2^ *σ*) of *TE* materials directly decides the output power of *TE* devices.^[^ ^7^ ^]^ However, TE materials with high *ZT* and *PF* are very rare.^[^ ^7^, ^8^ ^]^ While *ZT* is limited by thermal conductivity of *TE* material being intrinsic properties of the material, the *PF* depends on *S* and *σ*, which can be tuned. Therefore, growing investigations have focused on exploring potential *TE* materials and developing strategies to improve their *PF* and *ZT*.

Bulk Bi_2_Te_3_ exhibits supreme intrinsic *TE* properties at room temperature.^[^ ^9^ ^]^ Bi_2_Te_3_ received great attention since commercially available power generating *TE* modules rely on that material.^[^ ^10^ ^]^ *TE* films based on Bi_2_Te_3_ are also a hot topic currently.^[^ ^11^ ^]^ Manzi et al. demonstrated the use of plasma‐jet printed and electromagnetic field‐assisted printed colloidal *TE* Bi_2_Te_3_ nanoflakes.^[^ ^12^ ^]^ The material exhibit *PF* of 70 µWm^−1^ K^−2^ at room temperature. Chiba et al. prepared flexible films based on Bi_2_Te_3_ nanoplates and single‐walled carbon nanotubes via drop casting. The *PF* of 630 µWm^−1^ K^−2^ was achieved, indicating the high performance of flexible nanocomposite films.^[^ ^13^ ^]^ Ashfaq et al. demonstrated the that effectiveness of thermal evaporation as an approach for synthesizing the Sr doped Bi_2_Te_3_ thin films with *PF* of 1561 µWm^−1^ K^−2^ at room temperature.^[^ ^14^ ^]^ Moreover, confinement effects enhance *PF* significantly. In the case of Bi_38_Te_55_Se_7_ and Bi_15_Sb_29_Te_56_ nanowires, *PF*s of 2820 and 1750 µWm^−1^ K^−2^ at room temperature, respectively, were reported.^[^ ^15^ ^]^ However, this showed the impact of composition on the *PF*s in addition.

Besides, multilayer *TE* thin films have been developing in the past. Guo et al. fabricated Ni/Bi_0.5_Sb_1.5_Te_3_ *TE* material with multilayer structure, which exhibits the *PF* greater than 4 mWm^−1^ K^−2^ at 300 K.^[^ ^16^ ^]^ Liao et al. measured *TE* properties of spurted Sb/Bi–Sb–Te multilayer thin films after electrical stress. The *PF* of 1.36 mWm^−1^ K^−2^ was achieved at 603 K.^[^ ^17^ ^]^ Kim et al. deposited Sb_2_Te_3_/Bi_2_Te_3_ multilayer films via magnetron sputtering system, whose *PF* reached 493 µWm^−1^ K^−2^ at room temperature.^[^ ^18^ ^]^

Earlier, Mahan et al. proposed a special method to enhance the *PF* by combining materials exhibiting high electron concentration (e.g., metals) with semiconductors to introduce the distribution of carriers corresponding to the asymmetrical Fermi level.^[^ ^19^ ^]^ This resulted in a significant improvement of the *PF*. At the same time, the high interface density can also reduce thermal conductivity effectively, thus improving the overall *TE* properties.^[^ ^20^ ^]^ Thus, this article proposes the integration of W and Sb layers with Bi_2_Te_3_ layers in the form of Bi_2_Te_3_/Sb and Bi_2_Te_3_/W multilayer structures for combining their own advantages. Multilayer *TE* thin films commonly exhibit better *TE* properties than conventional *TE* materials due to the existence of interface optimization and band engineering within the films to refine the carrier transportation.^[^ ^21^ ^]^ Moreover, multilayer *TE* thin films can utilize the advantages of various properties from different materials, and by stacking these materials on top of each other, they can take full utilization of their respective advantages to improve the overall *TE* properties.^[^ ^21^ ^]^

*TE* performance is also related to the cycle of the layers. As reported,^[^ ^22^ ^]^ the layer number impact *TE* properties of *TE* materials. It was shown that the carrier concentration and mobility decreased, but the resistivity and Seebeck coefficient increased slightly and significantly, respectively, hereby increasing the power factor with increasing layer number. Consequently, keeping approximate same total thickness, the Bi_2_Te_3_/Sb and Bi_2_Te_3_/W multilayer thin films can result in a strong scattering effect compared with pure single‐layer Bi_2_Te_3_. The interlayers can reduce the mean free path of phonons, thus hindering phonon transport and decreasing the thermal conductivity. At the same time, the energy filtering effect of the semiconductor/metal interface can increase the Seebeck coefficient. Therefore, the mechanism of excellent *TE* properties of multilayer *TE* thin films is explored by measuring the *TE* properties and characterizing the microstructure of the multilayers. Overall, this work opens new frontiers in the development of *TE* materials with advanced properties by microstructure engineering.

## 2 Results and Discussions

### 2.1 Microstructure of Bi_2_Te_3_/Sb and Bi_2_Te_3_/W Multilayers

**Figure 1:**
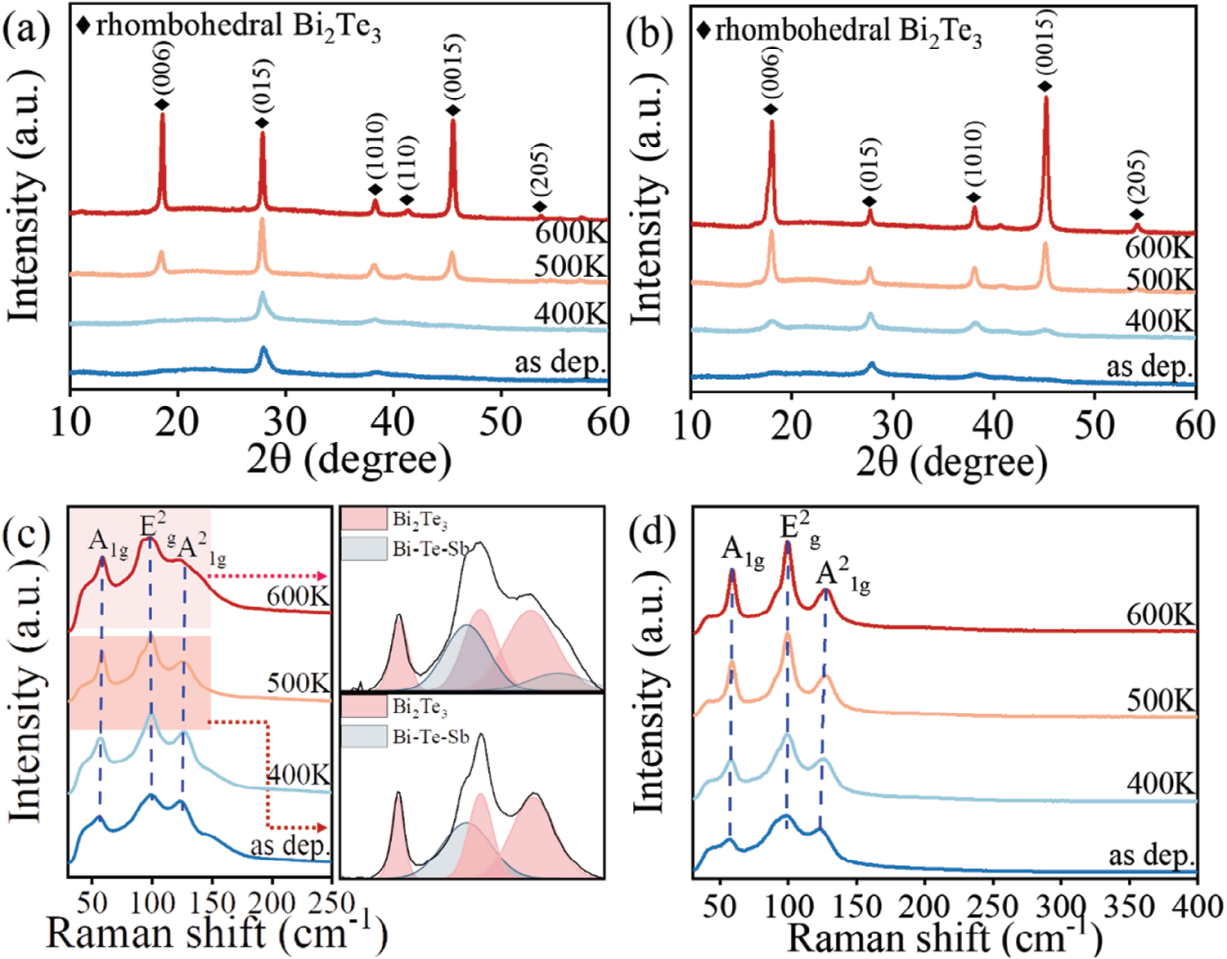
XRD patterns of as‐deposited and annealed a) Bi_2_Te_3_/Sb and b) Bi_2_Te_3_/W thin films. Raman spectra of as‐deposited and annealed c) Bi_2_Te_3_/Sb and d) Bi_2_Te_3_/W thin films.

**Figure** **1** shows XRD patterns and Raman spectra of as‐deposited and thermally annealed Bi_2_Te_3_/Sb and Bi_2_Te_3_/W thin films. In Figure 1a, the as‐deposited Bi_2_Te_3_/Sb thin film show diffraction peaks corresponding to rhombohedral Bi_2_Te_3_ (JCPDS NO. 8–27). This indicates the formation of nano crystallites of Bi_2_Te_3_ in the thin film at room temperature. After annealing to 500 K and 600 K, the intensity and number of peaks in XRD patterns are significantly increased. This shows grain growth in the Bi_2_Te_3_/Sb multilayer thin film. Figure 1b depicts XRD patterns of Bi_2_Te_3_/W multilayer thin films. All diffraction peaks also correspond to rhombohedral Bi_2_Te_3_ (JCPDS NO. 8–27). With temperature rise, diffraction peaks become sharper, while no diffraction peaks from W is observed. Thus, the W layers are amorphous in the thin films. The number and intensity of the diffraction peaks in the XRD patterns of the Bi_2_Te_3_/Sb and Bi_2_Te_3_/W multilayer films are different. After thermal heating, in line with single‐layer Bi_2_Te_3_ thin films (Figure S1, Supporting Information), Bi_2_Te_3_ is highly textured in the multilayer thin films with c‐axis oriented perpendicular to the substrate surface, although some other minor orientations are seen in the XRD patterns. However, Bi_2_Te_3_/Sb multilayers reveal additional strong (015) orientation. This suggests the impact of Sb and W layers on the microstructure in the multilayered samples.

Figure 1c,d shows Raman spectra of Bi_2_Te_3_/Sb and Bi_2_Te_3_/W multilayer thin films, respectively. The vibration modes A^1^_1g_, E^2^_g_, and A^2^_1g_ are related to Bi_2_Te_3_ and exhibit a small evolution with increasing of heating temperature.^[^ ^23^ ^]^ However, the E^2^_g_ of the Bi_2_Te_3_/Sb reveals larger shoulder at 400 K, while it splits into two peaks at 500 K. In addition, the peaks are broader in Bi_2_Te_3_/Sb multilayers than in Bi_2_Te_3_/W multilayers and single‐layer Bi_2_Te_3_ thin film (Figure S2, Supporting Information). This is a signature of intermixing with subsequent formation of amorphous Bi‐Te‐Sb phases. Taking the XRD and Raman patterns together, the peaks related to Bi_2_Te_3_ are the strongest peaks in all the samples. This shows that the crystallization of Bi_2_Te_3_ is dominant in the thin films. However, Sb tend to react with Bi_2_Te_3_ during heating, while W remained amorphous in the film (confirmed by TEM below).

**Figure 2:**
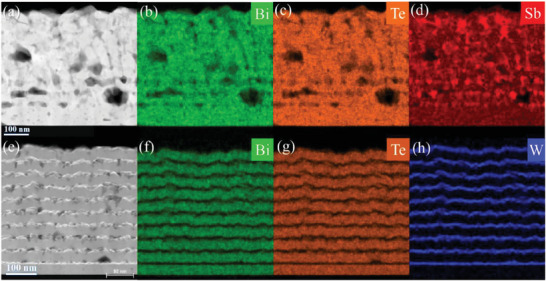
Cross‐sectional HAADF‐STEM images of a) Bi_2_Te_3_/Sb and e) Bi_2_Te_3_/W multilayers thermally heated at 600 K. Corresponding EDX elemental maps of Bi, Te and Sb are shown in (b), (c), (d), respectively, for the Bi_2_Te_3_/Sb and in (f), (g), (h), respectively, for the Bi_2_Te_3_/W. Dark areas surrounded by light/grey background in (a) and (e) correspond to cavities.

The effect of interdiffusion between the layers in Bi_2_Te_3_/Sb multilayer thin films was further revealed by nanoscale investigations using cross‐sectional TEM. **Figure** **2**a shows the HAADF‐STEM image of Bi_2_Te_3_/Sb thin film after thermal heating at 600 K. The corresponding EDX maps of Bi, Te, and Sb are shown in Figure 2b,c,d, respectively. HAADF images and EDX maps revealed vanishing of the multilayer structure, although some Sb‐rich layers are remained (Figure 2d). In addition, interdiffusion led to the precipitation of Sb‐rich Bi‐Te phases and the formation of large cavities in the Bi_2_Te_3_/Sb multilayers. Thus, the thin film after thermal heating represents an alloy consisting of Bi‐Te‐Sb phases and Sb‐rich Bi‐Te precipitates. The average composition of the Bi_2_Te_3_/Sb multilayers was measured to be 32.35 at.% of Bi, 47.65 at.% of Te and 20 at.% of Sb, while the average composition of Bi‐Te‐Sb phase being measured in specimen areas without Sb precipitates was identified to be 34.8 at.% (STD 0.75) of Bi, 50.7 at.% (STD 0.42) of Te and 14.5 at.% (STD 1.1) of Sb. The homogeneous area close to the substrate revealed the following composition 34.5 at.% of Bi, 50.8 at.% of Te and 14.7 at.% of Sb. Interestingly, the ratio of Bi to Te in the all cases is close to 1.5, showing the presence of Bi_2_Te_3_ as matrix. The composition of Sb‐rich Bi‐Te precipitates was identified to be 52.5 at.% (STD 8.5) of Sb, 20.8 at.% (STD 2.8) of Bi and 26.7 at.% of Te (STD 5.9).

Contrary to the Bi_2_Te_3_/Sb multilayers, the multilayer structure of Bi_2_Te_3_/W thin films remained unaffected after thermal heating at 600 K. EDX maps of Figure 2f,g depict well‐separated Bi_2_Te_3_ and W layers. Moreover, as in the previous case, cavities were also formed in the multilayers at the W/ Bi_2_Te_3_ interface (also Figure S3, Supporting Information). However, their distribution is more homogenous since they were formed at the interfaces between W and Bi_2_Te_3_, which are rough. Moreover, the size of the cavities is smaller, while their density is large compared to the Bi_2_Te_3_/Sb multilayers.

**Figure 3:**
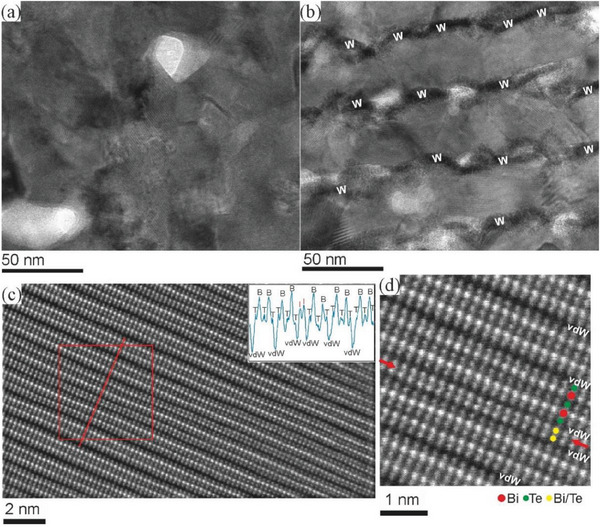
a,b) High‐resolution TEM images of Bi_2_Te_3_/Sb and Bi_2_Te_3_/W multilayers after thermally heating at 600 K. Cavities appear with bright contrast in the images. W marks W layers. c) Atomic‐resolution HAADF‐STEM micrograph of Bi_2_Te_3_ grain formed in the Bi_2_Te_3_/W. Insert depict line profiles extracted along the line shown in (c). T marks Te layers, B marks Bi layers and I marks Bi/Te bilayers. d) Magnified HAADF image of area in (c) marked by rectangle. The bilayer defect is marked by red arrows. vdW depicts van der Waals gaps in (c) and (d).

The grain sizes of Bi_2_Te_3_ in the Bi_2_Te_3_/W multilayers differ from the grain sizes of Bi_2_Te_3_ in the Bi_2_Te_3_/Sb multilayers. **Figure** **3** depicts high‐resolution TEM images of the multilayers. The grain boundaries in the Bi_2_Te_3_/W thin film are sharper and more defined compared to the Bi_2_Te_3_/Sb thin film. Although, the grain sizes of Bi_2_Te_3_ in the Bi_2_Te_3_/W multilayers are varied in lateral direction (from ≈25 to 100 nm), the sizes are restricted in the growth direction by the W layers. So, the thickness of the grains is limited to ≈35 nm, giving much narrow distribution of Bi_2_Te_3_ grain sizes. Contrary, the grain sizes of Bi_2_Te_3_ can be varied in all growing direction in the case of the Bi_2_Te_3_/Sb multilayers. Moreover, Bi_2_Te_3_ grains in the Bi_2_Te_3_/W multilayer showed high crystallinity. Figure 3c depicts atomic‐resolution HAADF‐STEM image of a part of c‐oriented Bi_2_Te_3_ grain (The full grain is shown in Figure S3, Supporting Information). The image reveals that Bi_2_Te_3_ crystal consists of building units with quintuple layers and the layers contain five atomic planes in the order of ‐Te‐Bi‐Te‐Bi‐Te. The building units are bonded together by weak van der Waals forces. In accordance with XRD measurements, the Bi_2_Te_3_ grains were formed with strong (001) texture (Figure S3, Supporting Information). Furthermore, stacking defects were also observed within of Bi_2_Te_3_ grains. The defects are confined between two atomic layers of Bi and Te and represent localized stacking faults (Figure 3c,d). The defects are typical for layered chalcogenide‐based compounds.^[^ ^24^ ^]^ Moreover, it was shown that such bilayer defects can enhance charge carrier mobility and thus the electrical conductivity of Bi_2_Te_3_ (also *TE* material) as well as they can reduce lattice thermal conductivity by inhibiting the phonon transport.^[^ ^25^ ^]^

### 2.2 TE Properties of Bi_2_Te_3_/Sb and Bi_2_Te_3_/W Multilayers

**Figure 4:**
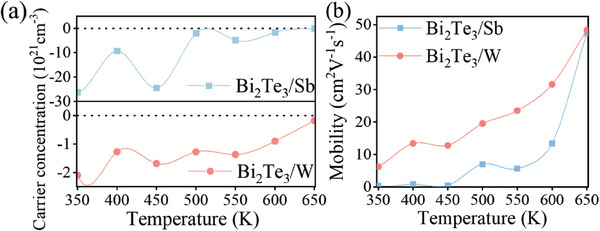
a) Carrier concentration and b) mobility of the as‐deposited Bi_2_Te_3_/Sb and Bi_2_Te_3_/W films as a function of annealing temperature.

**Figure** **4** shows the carrier concentration and mobility of Bi_2_Te_3_/Sb and Bi_2_Te_3_/W multilayers as a function of annealing temperature. The negative values of carrier concentration demonstrate n–type conduction, indicating that electrons are the major carriers. The carrier concentration in Bi_2_Te_3_/Sb (Figure 4a) exhibits fluctuations to some degree during annealing before 450 K. This can be due to compositional changes occurring on annealing as revealed by TEM measurements. After annealing at temperatures of 450 and 500 K, the carrier concentration of Bi_2_Te_3_/Sb thin films decreased significantly from −2.455 × 10^22^ to −2.091 × 10^21^ cm^−3^. Then, the carrier concentration tends to stabilize from 500–650 K. This implies that the reaction induced by interdiffusion between Bi_2_Te_3_ and Sb has been almost completed. However, the carrier concentration in Bi_2_Te_3_/W multilayers remains constant during annealing, which is due to high quality Bi_2_Te_3_ grains. Unlike carrier concentration, the mobility (Figure 4b) of the thin films shows a continuous increasing trend with temperature. It can be ascribed to that the gradual crystallization of the multilayers leads to the high mobility. This is beneficial for the enhancement of electrical conductivity and the achievement of excellent *TE* properties.

**Figure 5:**
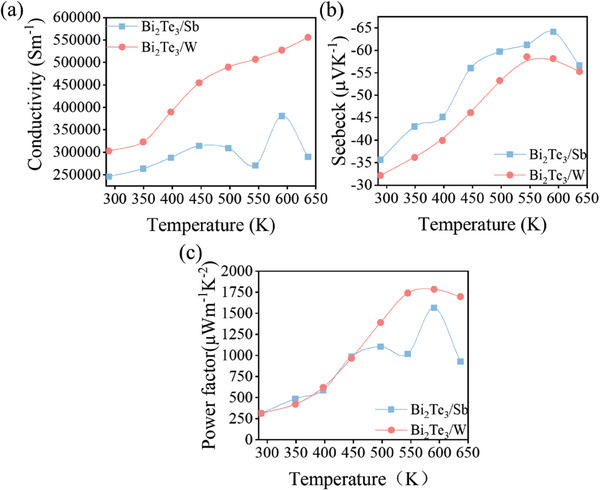
Temperature‐dependent a) electrical conductivity, b) Seebeck coefficient, and c) PF for Bi_2_Te_3_/Sb and Bi_2_Te_3_/W multilayers.

**Figure** **5** displays the evolution of electrical conductivity, Seebeck coefficient and *PF* of Bi_2_Te_3_/Sb and Bi_2_Te_3_/W multilayers as a function of heating temperature. Figure 5a shows that all samples exhibit extremely high electrical conductivity. According to Figure 5a, Bi_2_Te_3_/Sb possesses the lowest electrical conductivity of 2.5 × 10^5^ S m^−1^ at room temperature. With the heating temperature increasing, the electrical conductivity increased slightly due to interdiffusion. While the electrical conductivity of Bi_2_Te_3_/W exhibits a significantly increasing trend with the highest value of 5.6 × 10^5^ S m^−1^ at 650 K. This can be ascribed to the controllable multilayer structure of Bi_2_Te_3_/W with the confined Bi_2_Te_3_ grains in good crystallinity. The overall electrical conductivity increases with temperature, indicating semiconductor behavior of the samples. *TE* materials with lower internal resistance could dissipate less energy while operating, which is critical in promoting the energy conversion efficiency.^[^ ^26^ ^]^

Figure 5b depicts the temperature‐dependent variation of Seebeck coefficient. The coefficients are negative for all samples, which means that the thin films are n‐type semiconductors. The Seebeck coefficient increases significantly as temperature rising, where Bi_2_Te_3_/Sb exhibits the highest Seebeck coefficient of −64 µVK^−1^ at 600 K. While the crystallization of Bi_2_Te_3_/Sb multilayers can lead to the increase in the carrier concentration, the crystal interfaces and cavities can scatter carriers and reduce mobility, which leads to the low electrical conductivity and high Seebeck coefficient. Above 600 K, the Seebeck coefficient decreases due to the bipolar effect.^[^ ^27^ ^]^ Figure 5c represents the calculated *PF*s. The overall *PF* increases with annealing temperature. Bi_2_Te_3_/W exhibits the highest *PF* of 1785 µWm^−1^ K^−2^ at 600 K. Beyond the expectation, the results show that the electrical conductivity and Seebeck coefficient could increase at the same time with temperature rise. Though the Seebeck coefficient is low, the extremely high electrical conductivity contributes to much higher *PF* of Bi_2_Te_3_/W multilayers than Bi_2_Te_3_/Sb multilayers and single‐layer Bi_2_Te_3_ thin film (Figure S4, Supporting Information), indicating the effect of carrier transport modulation within the multilayer nanocomposite films. This indicates that the samples can conquer the intrinsic contradictory relationship between the electrical conductivity and Seebeck coefficient, which contributes considerably to such high *PF*.

Moreover, in Figure 5b, there is an upward trend observed in S‐T plot, coinciding with an increase in electrical conductivity (Figure 5a). Two cases are warrant for discussion: Bi_2_Te_3_/Sb multilayers and Bi_2_Te_3_/W multilayers. In the first case, Figure 5b reveals an upward trend in Seebeck coefficient at temperatures above 600 K, concurrently with a decrease in electrical conductivity (Figure 5a). This trend may be attributed to additional microstructural and compositional changes occurring at higher temperatures. In the second case, Figure 5b shows a slight decrease in Seebeck coefficient at temperatures higher than 550 K, while electrical conductivity continues to rise (Figure 5a). Given that only the Bi_2_Te_3_ phase is formed in Bi_2_Te_3_/W multilayers, the formation of more defects at higher temperatures, including larger voids, might explain this behavior. Although, in general, nanoscale voids reduce the thermal conductivity of *TE* materials, positively influencing the *ZT* value, larger voids can have the opposite effect. Similar trends in Seebeck coefficient and electrical conductivity were observed in Ag‐Mn‐Sb‐Te and Mg–Zn–Sb alloy.^[^ ^28^, ^29^ ^]^ However, the decrease in Seebeck coefficient did not significantly impact the overall *ZT* value in these cases, as the formation of nanoscale voids contributed to the reduction of thermal conductivity.^[^ ^29^ ^]^

### 2.3 Discussion on the Enhancement of PFs in Bi_2_Te_3_/Sb and Bi_2_Te_3_/W Multilayers

High electrical conductivity at room temperature can be attributed to the presence of high intrinsic conductive layers of Sb and W. With temperature rise, however, interdiffusion between the layer in the Bi_2_Te_3_/Sb multilayers resulted in compositional changes with subsequent formation of Bi‐Te‐Sb and Sb‐rich Bi‐Te grains as well as cavities. The formation of new grains and grain boundaries lead to a reduction in carrier concentration (Figure 4a), which results in the enhancement of Seebeck coefficient.^[^ ^30^ ^]^ However, the Bi_2_Te_3_/W multilayers are more stable, and the multilayer structure is maintained after thermal heating. Moreover, confinement of Bi_2_Te_3_ grains between W layers and their high crystalline quality led to higher electrical conductivity with temperature rise, while the cavities and bilayer defects will result in low thermal conductivity, which is beneficial for *ZT* value. The confined Bi_2_Te_3_ crystals cause insufficient grain boundaries for effectively blocking carriers, which contribute to lower Seebeck coefficient compared to the Bi_2_Te_3_/Sb multilayers. It should be noted that the successive W layers caused the short circuit in the test, which also caused the measured Seebeck coefficient to be lower than the actual value. The carrier transportation in the sample can be divided into two parts. In the direction parallel to the layers, the carrier transportation is little hampered due to the strong conductive W interlayers, while confined Bi_2_Te_3_ grains of high‐quality lead to the increase of grain boundaries, which are also beneficial for filtering carriers. In the direction vertical to the layers, the multilayer structure would provide more interfaces and opportunities for blocking and filtering. The contact interfaces between semiconductor and metal would induce Schottky barrier,^[^ ^31^ ^]^ which is critical for filtering low‐energy carriers and regulating carrier concentration in a reasonable scale. The combination of semiconductor and metal can modify electronic properties because of the coupling and synergistic effects. The interfaces of different energy band structures can optimize the carrier transportation, even refine the overall *TE* properties. In conclusion, the interlayers of Sb and W behave differently in boosting the overall *TE* properties. The phase boundary modulation of Bi_2_Te_3_/Sb and the multilayer interface engineering of Bi_2_Te_3_/W allow the samples to conquer the intrinsic contradiction between the electrical conductivity and the Seebeck coefficient. Thus, the synchronously elevated electrical conductivity and Seebeck coefficient together facilitate excellent *PF*. This provides a feasible way for investigation and fabrication of *TE* materials with high properties.

## 3 Conclusion

Bi_2_Te_3_/Sb and Bi_2_Te_3_/W multilayer nanocomposite *TE* thin films were designed by using magnetron sputtering. The films are modified in terms of microstructure to optimize their *TE* properties. Because of the multilayer structure, the intrinsic contradictory relationship between electrical conductivity and Seebeck coefficient can be overcome. As a result, the multilayer samples exhibit very high electrical conductivity, which contributes to reduce energy dissipation while generating electricity. The experimental outcomes showed that the microstructure of Bi_2_Te_3_/Sb and Bi_2_Te_3_/W thin films can rationally regulate the carrier concentration and filter low‐energy carriers to enhance the electrical conductivity and Seebeck coefficient at the same time. With the simultaneous elevation of electrical conductivity and Seebeck coefficient, Bi_2_Te_3_/W multilayers exhibits the highest *PF* of 1785 µWm^−1^ K^−2^ at 600 K. Overall, this work offers a novel approach to overcome the intrinsic contradictions of *TE* materials and provides the strategies for designing advanced novel multilayer/nanocomposite *TE* thin films with enhanced properties.

## 4 Experimental Section

Multilayer Bi_2_Te_3_/Sb and Bi_2_Te_3_/W thin films were deposited on quartz and SiO_2_/Si(100) substrates through magnetron sputtering at room temperature. Bi_2_Te_3_, Sb and W targets were all installed on radio frequency targets, and the power for sputtering of each target was set to 50, 50, and 40 W, respectively. The Bi_2_Te_3_, Sb and W targets target were alternately turned on and switched off. 10 layers of Bi_2_Te_3_ and 10 layers of W or Sb were deposited in this way. In each cycle, for the growth of multilayer Bi_2_Te_3_/Sb thin films, the Bi_2_Te_3_ target was first switched on for 111 s then switched off, while the Sb target was switched on for 40 s then switched off. Similarly, for the deposition of multilayer Bi_2_Te_3_/W thin films, in one cycle the alternate switching on duration for Bi_2_Te_3_ and W target was 111 and 180 s, respectively. The pressure during sputtering was kept at 4 × 10^−6^ Pa, and the Ar flow rate was fixed to 60 mL min^−1^. After the depositions, the substrates were cut into small pieces to facilitate further processing. The samples fabricated at room temperature were put into the vacuum oven for annealing. The thin films were annealed at 450, 500, 550, 600, and 650 K with N_2_ as shielding gas for 10 min. The Seebeck coefficient and electrical conductivity of the thin films were measured using in‐situ thin film TE parameter testing system (SBA458, NETZSCH Instrument Manufacturing Company, Germany) with the measurement error of less than 7% and 10%, respectively. In order to investigate the crystallization and atomic bonding in the films, X‐ray diffraction (XRD, D2 Phaser, Bruker, Germany) and Raman spectrometry were utilized to analyze the thin films, respectively. The maximum allowable error range of XRD results for angle and intensity is ≤ ± 5%, while the offset of the peaks in Raman results is ≤ ± 0.5 cm^−1^, and the accuracy of the peak intensity is ≤ ± 5%. The carrier concentration and mobility of the crystalline films were measured using conventional Hall measurements (HP–5500C, Nanometrics, USA). Advanced transmission electron microscopy (TEM) investigations were carried out via a probe Cs‐corrected Titan^3^ G2 60–300 microscope equipped with high‐angle annular dark‐field (HAADF) detector and Super‐X EDX system. During the experiments a probe forming aperture of 25 mrad was utilized. The elemental maps were obtained in scanning TEM (STEM) mode using 120 pA beam current. TEM was operated at 300 kV accelerating voltage. TEM specimens for the TEM investigations were prepared by focused ion beam (FIB) milling.

## Conflict of Interest

The authors declare no conflict of interest.

## Author Contributions

G.‐X.W. and A.L. contributed equally to this work. F.‐Z.M. prepared the multilayer films. Y.‐Q.C. carried out the thermoelectric measurements. A.L. performed the microstructural characterizations and atomic arrangement analysis. X. S. provided technical assistance. F.‐Z.M. and G.‐X.W. wrote the manuscript with the contributions from A.L. and X.S. All authors discussed the results and commented on the manuscript.

## Supporting information

Supporting Information

## Data Availability

The data that support the findings of this study are available from the corresponding author upon reasonable request.
