# Supplementary material for: Boosting Thermoelectric Performance of Bi2Te3 Material by Microstructure Engineering
Source: Adv Sci (Weinh). 2023 Dec 7;11(6):2308056. doi: 10.1002/advs.202308056 (PMC10853752; doi:10.1002/advs.202308056)
Supplement: Supplementary file 1 — Supporting Information [file ADVS-11-2308056-s001.pdf]

ADVANCED SCIENCE
Open Access

Supporting Information

for *Adv. Sci.*, DOI 10.1002/advs.202308056

Boosting Thermoelectric Performance of Bi_2Te_3 Material by Microstructure Engineering

Guoxiang Wang, Fanzheng Meng, Yingqi Chen, Andriy Lotnyk* and Xiang Shen**

Supplementary Information For

Boosting thermoelectric performance of Bi_2Te_3 material by microstructure engineering

Guoxiang Wang[a*], Fanzheng Meng[a], Yingqi Chen[a], Andriy Lotnyk[a,b,*], Xiang Shen[a,c*]

[a]*Laboratory of Infrared Materials and Devices, The Research Institute of Advanced Technologies, Ningbo University, Ningbo, Zhejiang 315211, China.*

[b]*Leibniz Institute of Surface Engineering (IOM), Permoserstr. 15, D-04318 Leipzig, Germany*

[c]*Institute of Ocean Engineering, Ningbo University, Ningbo, Zhejiang 315211, China*

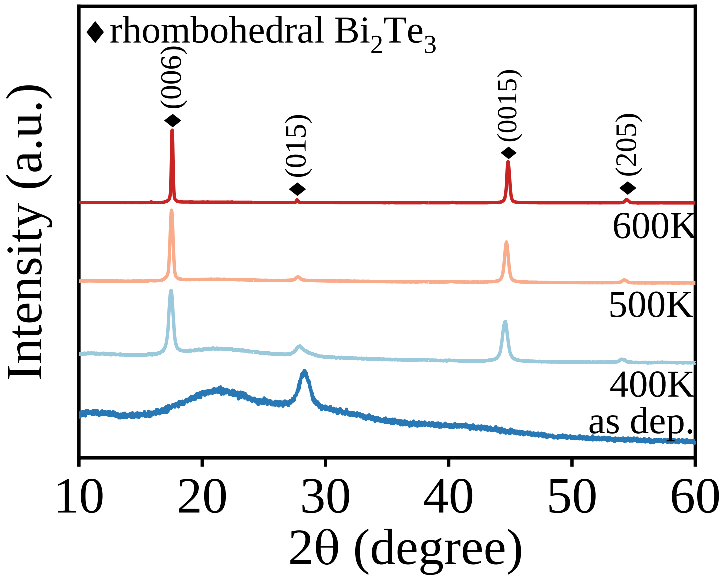

Figure S1. XRD patterns of as-deposited and annealed Bi_2Te_3 films.

*Corresponding authors.

E-mail addresses: wangguoxiang@nbu.edu.cn (G.X. Wang), andriy.lotnyk@iom-leipzig.de (A. Lotnyk) and shenxiang@nbu.edu.cn (X. Shen)

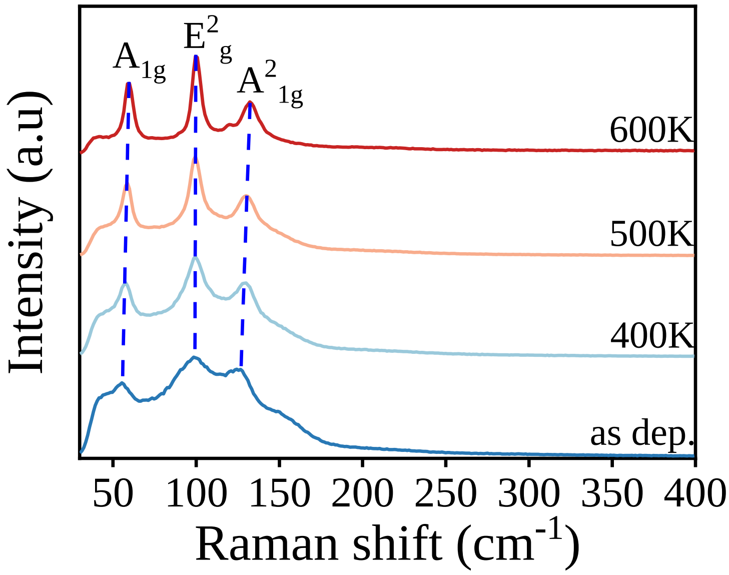

Figure S2. Raman spectra of as-deposited and annealed single-layer Bi_2Te_3 thin films

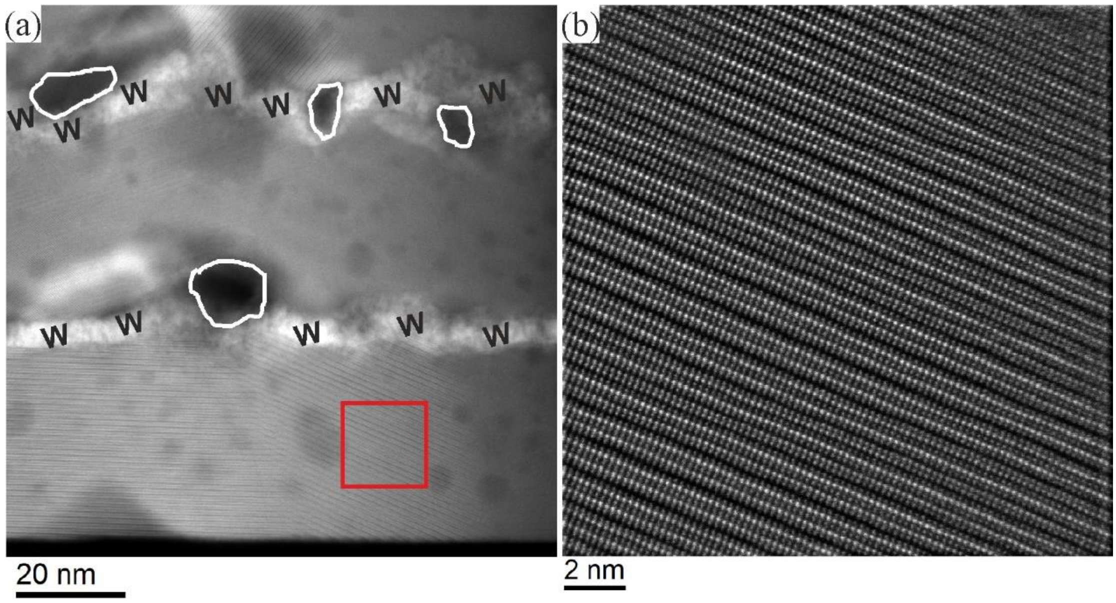

Figure S3. Full area of a grain shown in Fig. 3(c) of the main text. (a) HAADF-STEM image of Bi_2Te_3/W multilayers after thermally heating at 600 K. Cavities appear with dark contrast as marked in the image. W marks W layers. (b) Atomic-resolution HAADF-STEM image of area marked by rectangle in (a). One part of the micrograph is shown in Fig. 3(c).

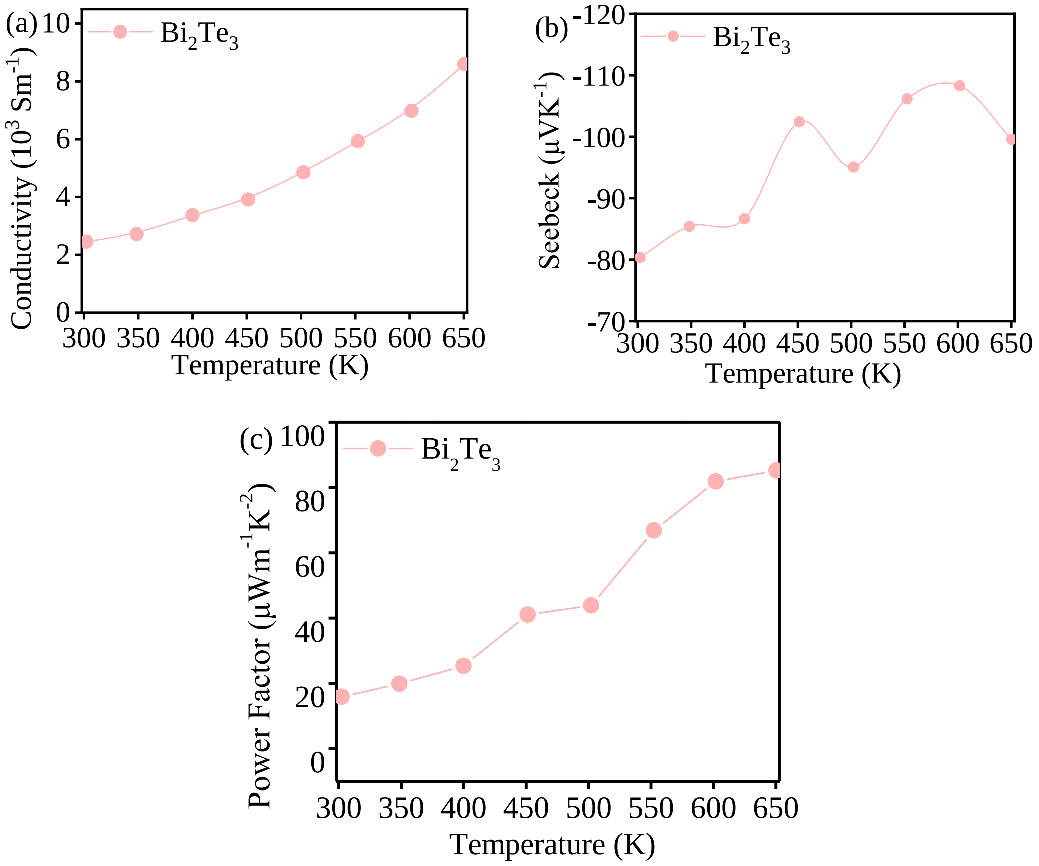

Figure S4. (a) Conductivity, (b) Seebeck coefficient, and (c) PF of single-layer Bi_2Te_3 thin films as functions of temperature.
